# Circadian variation in acute myocardial infarct size assessed by cardiovascular magnetic resonance in reperfused STEMI patients

**DOI:** 10.1016/j.ijcard.2016.12.030

**Published:** 2017-03-01

**Authors:** Heerajnarain Bulluck, Jennifer Nicholas, Gabriele Crimi, Steven K White, Andrew J Ludman, Silvia Pica, Claudia Raineri, Hector A Cabrera-Fuentes, Derek Yellon, Jose Rodriguez-Palomares, David Garcia-Dorado, Derek J Hausenloy

**Affiliations:** a The Hatter Cardiovascular Institute, Institute of Cardiovascular Science, University College London, UK; b The National Institute of Health Research University College London Hospitals Biomedical Research Centre, UK; c London School Hygiene and Tropical Medicine, London, UK; d Royal Devon and Exeter Hospital NHS Foundation Trust, Exeter, UK; e Struttura Complessa Cardiologia, Fondazione Istituto Di Ricovero e Cura a Carattere Scientifico (IRCCS) Policlinico San Matteo, Pavia, Italy; f Struttura Complessa Cardiologia, Azienda Ospedaliera SS: Antonio e Biagio, Alessandria, Italy; g Cardiology Department, Vall d'Hebron Hospital, Universitat Autónoma de Barcelona, Barcelona, Spain; h Institute of Biochemistry, Medical School, Justus-Liebig-University, Giessen, Germany; i National Heart Research Institute Singapore, National Heart Centre Singapore, Singapore; j Cardiovascular and Metabolic Disorders Program, Duke-National University of Singapore, Singapore

**Keywords:** ST-segment elevation myocardial infarction, Circadian rhythm, Myocardial infarct size, Cardiovascular magnetic resonance

## Abstract

**Objective:**

Clinical studies using serum cardiac biomarkers to investigate a circadian variation in acute myocardial infarct (MI) size in ST-segment elevation myocardial infarction (STEMI) patients reperfused by primary percutaneous coronary intervention (PPCI) have produced mixed results. We aimed to investigate this phenomenon using acute MI size measured by cardiovascular magnetic resonance (CMR).

**Methods:**

Patient-level data was obtained from 4 randomized controlled trials investigating the MI-limiting effects of cardioprotective therapies in this pooled analysis. The primary analysis was performed in those patients with no pre-infarct angina; duration of ischemia > 60 min and < 360 min; Thrombolysis In Myocardial Infarction (TIMI) flow pre-PPCI ≤ 1; TIMI flow post-PPCI 3; and no collateral flow.

**Results:**

169 out of 376 patients with CMR data met the inclusion criteria for the primary analysis. A 24-hour circadian variation in acute MI size as a % of the area-at-risk (%AAR), after adjusting for confounders, was observed with a peak and nadir MI size in patients with symptom onset between 00:00 and 01:00 and between 12:00 and 13:00 respectively (difference from the average MI size 5.2%, 95%CI 1.1–9.4%; *p* = 0.013). This was associated with a non-significant circadian variation in left ventricular ejection fraction (LVEF) (difference from the average LVEF 5.9%, 95%CI − 0.6–2.2%, *p* = 0.073). There was no circadian variation in MI size or LVEF in the whole cohort.

**Conclusions:**

We report a circadian variation in acute MI size assessed by CMR in a subset of STEMI patients treated by PPCI, with the largest and smallest MI size occurring in patients with symptom onset between 00:00 and 01:00 and between 12:00 and 13:00 respectively.

## What is already known about this subject?

Several studies [1], [2], [3], [4], [5], [6], [7] have shown a circadian variation in myocardial infarct (MI) size by cardiac enzymes according to the time of onset of symptoms but the literature is divided regarding the timing of the peak MI size. Some studies have shown that peak MI size occurs in patients with symptom onset between midnight and 06:00 [3], [6], [7] while others have shown peak MI size occurring in those with symptoms onset between 06:00 and noon [1], [4], [5].

## What does this study add?

In this study we used cardiac magnetic resonance (CMR), which is the gold standard method for assessing MI size, and we adjusted for the area-at-risk (AAR) and confounders. We confirmed using CMR that in a selected group of patients a circadian pattern in MI size exists and that peak MI size occurred in those with symptom onset between midnight and 06:00. This was associated with a non-significant circadian variation in acute left ventricular ejection fraction (LVEF).

## How might this impact on clinical practice?

The circadian dependence in acute MI size was only observed in a subset of patients entering RCTs. This needs to be taken into account to make sure that the patients are adequately balanced according to the time of symptom onset when designing future RCTs aiming to reduce MI size. Whether this would impact on the effectiveness of the cardioprotective therapies needs to be assessed in future larger studies.

## 1 Introduction

The circadian rhythm has been shown to modulate cardiovascular physiology, impacting on parameters such as heart rate and blood pressure [8], [9], [10], [11], [12] via the expression of a circadian clock gene in the heart [13], [14]. A circadian oscillation has also been shown to impact on the expression of some proteins in the pro-survival pathways [15], and the susceptibility of the myocardium to acute ischemia/reperfusion injury (IRI), following myocardial infarction (MI) in mammalian hearts [16], [17].

Durgan et al. [18] reported the existence of circadian dependence in MI size according to the time of day in a murine model of acute myocardial IRI. Since then, several groups have investigated whether a circadian variation also exists in ST-segment elevation myocardial infarction (STEMI) patients but the results have been conflicting, both in terms of the timings for the peak and the nadir of acute MI size [1], [2], [3] and whether the phenomenon exists at all in humans [19]. Clinical studies investigating the circadian variation in acute MI size have assessed irreversible myocardial injury using serum cardiac biomarkers such as creatine kinase (CK) [1], [2], [3], [6], CK-MB [5], and troponin I [1], [20], and have determined the effects on left ventricular ejection fraction (LVEF) by echocardiography [5]. Cardiovascular magnetic resonance (CMR) is considered the gold standard for acute MI size quantification [21], [22], [23], the measurement of LV volumes and LVEF [23], [24], and can also provide information on the area-at-risk (AAR) [25], [26], but so far it has not been used to investigate the circadian variation in acute MI size in STEMI patients. If a circadian dependence in acute MI size is also observed by CMR, this would be an important factor to be taken into account in the design of future randomized controlled trials (RCTs) aiming to reduce MI size.

Therefore the aim of the current study was to investigate whether there is a circadian variation in acute MI size measured by CMR in STEMI patients reperfused by primary percutaneous coronary intervention (PPCI). In order to minimize the heterogeneity of the patient population and to remove potential confounders for acute MI size, the primary analysis was performed using a pre-defined subset of STEMI patients meeting specific criteria as previously described by Reiter et al. [3]

## 2 Methods

### 2.1 Study population

This was a pooled analysis of patient-level data obtained from 4 published randomized controlled trials investigating the benefit of cardioprotective therapies in STEMI patients treated by PPCI, and using acute MI size by CMR as an end-point (Table 1). The individual methods and results for each study have been published previously [27], [28], [29], [30] and all studies were conducted in accordance to the Declaration of Helsinki.

### 2.2 Outcomes

The main outcome of interest was acute MI size by CMR, expressed as a % of the AAR (%AAR) and LVEF. The primary exposure of interest in all analyses was the time of day, defined as the time of onset of MI symptoms and recorded to the nearest minute. The primary analysis examined the effect of time as a continuous measure, using a periodic sinusoidal function for the circadian cycle by time of symptom onset, as per the methods of Reiter et al. [3] in a selected group of patients defined by the following criteria [3]: no pre-infarct angina (which may have inadvertently preconditioned the patients and reduced MI size); duration of ischemia > 60 min and < 360 min; Thrombolysis In Myocardial Infarction (TIMI) flow pre-PPCI 0 or 1; TIMI flow post-PPCI 3; and no retrograde filling of the distal vessels (Rentrop grade 0). A secondary analysis was performed in the total unselected cohort of patients.

### 2.3 Statistical analysis

All analysis was performed using Stata version 12.1 (StataCorp, College Station, Texas). The period of the circadian cycle was defined in advance by converting time of onset into radians using the appropriate scale. For example, to model a 24-hour period in the circadian cycle the conversion is:

(1) $$r=2\pi \frac{\mathrm{time of onset}}{24}$$

Then the sinusoidal function for infarct size (*y*) is expressed as follows:

(2) $$y=\alpha +\text{βsin}\left(r+\omega \right)$$

The parameters to be estimated were: α representing the mean infarct size; β representing the amplitude of the rhythm; ω representing the phase of the curve, which determines when in the circadian cycle that the maximum and minimum infarct sizes occur.

The model in Eq. (2) was fitted using trigonometric linear regression as outlined by Cox [31]. A 24-hour period for circadian rhythm was assumed for the initial model and then further terms were included for sinusoidal function with a 12-hour period. Following fitting of the model using trigonometric linear regression, the delta method was used to provide Wald test *p*-values and 95% confidence intervals. Analysis was also performed by grouping patients into quartiles by time of day of onset of symptoms (00:00 to 05:59, 6:00 to 11:59, 12:00 to 17:59, 18:00 to 23:59).

The following confounders for MI size were adjusted for in the primary analysis: originating study, intervention (control, erythropoietin, remote ischemic conditioning upper limb or lower limb, adenosine); baseline demographic characteristics such as age and gender; onset to balloon time; risk factors (smoking; diabetes; hypertension; dyslipidemia; family history of coronary artery disease); and infarct related artery. For the secondary analysis, adjustment was made for these additional confounders: pre-infarct angina; TIMI flow pre-PPCI; presence of collateral flow; and TIMI flow post-PPCI.

## 3 Results

**Fig. 1:**
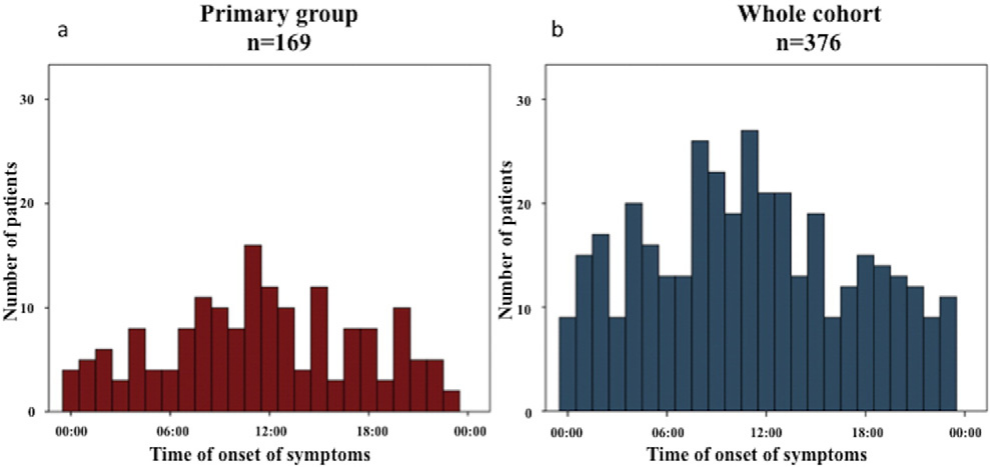
Distribution of patients according to time of onset of symptoms: (a) primary group of 169 patients; (b) whole cohort. As expected, significantly more patients were recruited in these RCTs with time of symptom onset occurring during daytime.

Acute CMR data were available in 376 patients and 169 patients met the inclusion criteria for the primary analysis. Fig. 1 shows the distribution of patients in the primary analysis and for the whole cohort according to the time of symptom onset. Data on the baseline demographics, angiographic and CMR findings of the whole cohort is provided in Table 2. Table 3 provides further details between those not included (n = 207) and included (n = 169) in the primary analysis. Apart from the selection criteria used, the other notable differences between patients in the primary analysis when compared to those not included in the primary analysis were: more patients from Ludman et al. [27] and White et al. [29] and less patients from Garcia-Dorado [30]; more males; less smokers; and more right coronary artery territory MI and less circumflex territory MI.

### 3.1 AAR and circadian rhythm

In the unadjusted analysis, there was evidence of a 24-hour cycle circadian variation in AAR with the difference between natural log of average and the peak value estimated as 0.07 (95% CI 0.01 to 0.12, *p* = 0.027). After adjusting for confounders, there was no longer evidence of a circadian rhythm in the AAR with the difference between natural log of average and peak values of 0.03 (95% CI − 0.02 to 0.08, *p* = 0.27). When a 12-hour cycle of circadian dependence was considered, there was no evidence of a circadian variation in AAR.

### 3.2 Primary analysis

**Fig. 2:**
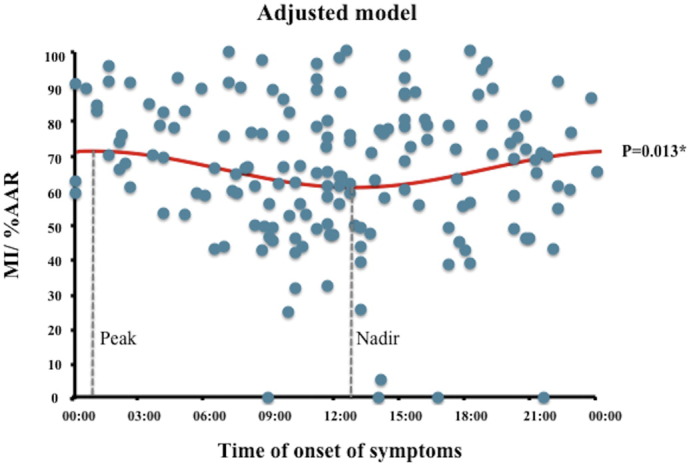
Circadian variation in MI size as a percentage of the AAR in the subset of patients in the primary analysis. The peak MI size/%AAR occurred between 00:00 and 01:00 and the nadir occurred between 12:00 and 13:00. * denotes statistical significant with a *p* value of < 0.05.

**Fig. 3:**
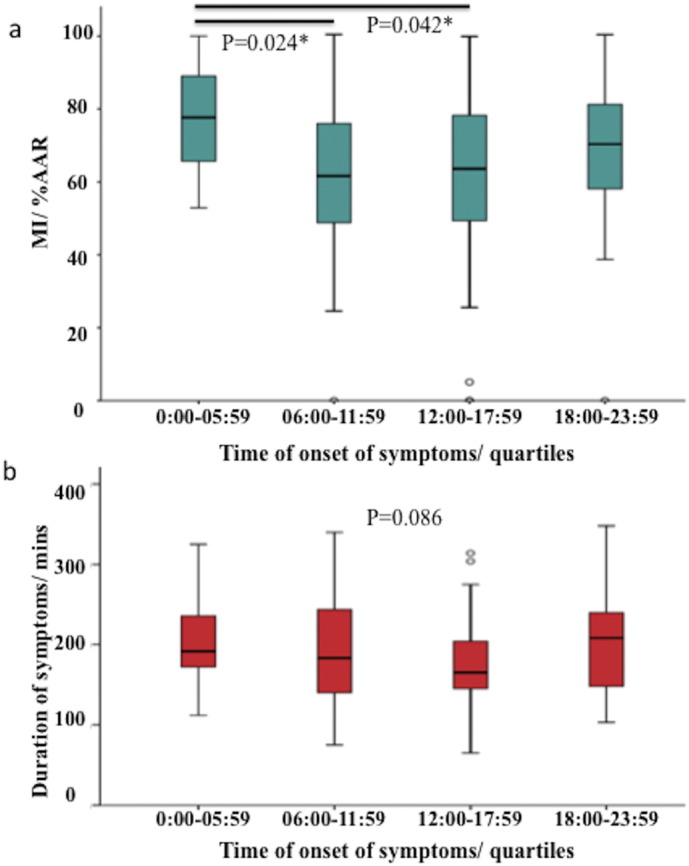
(a) MI size as a percentage of the AAR in the subset of patients in the primary analysis divided into quartiles of time of onset of symptoms; (b): comparison of duration of symptoms according to quartiles of time of onset of symptoms. The largest MI size occurred in the 00:00 to 05:59 group and there was no difference in the duration of symptoms in the 4 quartiles. * denotes statistical significant with a *p* value of < 0.05.

There was evidence for a 24-hour cycle in MI size as %AAR after adjusting for confounders with the maximum MI size being 5.2% larger than the average MI size (95 CI 1.1 to 9.4%, *p* = 0.013) (Fig. 2). The MI size was the largest for symptom onset between 00:00 and 01:00, and the smallest for symptom onset between 12:00 and 13:00. There was no evidence for a 12-hour circadian rhythm in MI size expressed as %AAR (for adjusted model: maximum MI size being 3.3% larger than the average, 95% CI − 0.6 to 7.2%, *p* = 0.10). When patients were grouped into quartiles by time of day of onset of symptoms (00:00 to 05:59, 6:00 to 11:59, 12:00 to 17:59, 18:00 to 23:59), the largest MI size occurred in the 00:00 to 05:59 group (Fig. 3a) and there was no significant difference in the duration of symptoms by quartiles to account for that (Fig. 3b).

There was a non-significant circadian variation in LVEF in this subgroup both before and after adjusting for confounders (unadjusted model: difference between average and peak LVEF 6.0%, 95% CI − 0.6 to 12.6%, *p* = 0.074; adjusted model: difference between average and peak LVEF 5.9%, 95% CI − 0.6 to 12.23%, *p* = 0.073).

### 3.3 Secondary analysis — whole cohort

When MI size was expressed as %AAR, there was no evidence of a 24-hour cycle of circadian dependence for MI size (%AAR) both in the unadjusted model (difference between the average and peak value: 1.8%, 95% CI − 1.2 to 4.9%; *p* = 0.24) and after adjusting for confounders (difference between the average and peak value: 2.7%, 95% CI − 0.1 to 5.5%; *p* = 0.06). When a 12-hour cycle of circadian dependence was considered, there was no evidence of a circadian variation in MI size expressed as a percentage of the AAR.

When considering LVEF, there was no evidence of a 24-hour cycle of circadian variation both in the adjusted and unadjusted analysis. When a 12-hour circadian cycle was considered, there was evidence of a circadian variation in LVEF in the unadjusted analysis (difference between the average and the peak value was estimated as 2.2%, 95% CI 0.7 to 3.8%; *p* = 0.006). After adjusting for confounders, there was a non-significant difference in circadian variation in LVEF (difference between the average and peak value: 1.1%, 95% CI − 0.2 to 2.5%; *p* = 0.092).

## 4 Discussion

Our study shows that in a selected group of 169 patients in whom confounders (including duration of symptoms) for acute IRI and MI size were minimized, there was evidence of a 24-hour circadian variation in acute MI size after adjusting for the AAR. The greatest acute MI size occurred in STEMI patients with symptom onset between 00:00 and 01:00 and the nadir was in those with symptom onset between 12:00 and 13:00. The mean difference between the peak and nadir MI size (%AAR) was 10.4%, corresponding to 3.5% of the LV. When divided into quartiles, the largest MI size occurred in the 00:00 to 05:59 group. This 24-hour circadian variation in acute MI size was associated with a non-significant change in LVEF. However, there was no evidence of a circadian variation in acute MI size in the unselected cohort of 376 STEMI patients, after accounting for the AAR and confounding factors.

Following the initial work by Durgan et al. [18] who showed that acute MI size was largest in the sleep-to-wake transition and this phenomenon was abolished in cardiomyocyte-specific circadian clock mutant rodents [18], the clinical studies have shown inconsistent results as summarized in Table 4. Our findings are in keeping with Reiter et al. [3] that showed a peak MI size occurring at 01:00. Furthermore, 2 multicentre studies (Seneviratna et al. [6] and Mahmoud et al. [7]) showed that the peak MI size occurred in those with symptom onset between midnight and 06:00 and this was confirmed in our study. On the other hand, 3 other studies (Suarez-Barrientos et al. [1], Fournier et al. [4] and Ari et al. [5]) have shown that the peak MI size occurred in those with symptom onset between 06:00 and noon. However, Suarez-Barrientos et al. [1] used a 12-hour cycle and a second peak was also observed in the 18:00 to midnight group. Fournier et al. [2] subsequently reported in a multi-centre Swiss study of 6233 patients [2] that the peak CK occurred in patients with symptom onset at 23:00 and the risk of death was the highest for those with symptom onset at 00:00. In terms of clinical outcomes, Seneviratna et al. [6] also showed a similar pattern in the incidence of acute heart failure and 1-year mortality rate whereas Mahmoud et al. [7] did not find the time of symptom onset to be a significant predictor of 1-year mortality in an equally large number of patients. In a multicentre international collaborative study of 1099 patients from China, Scotland and Italy, Ammirati et al. [19] showed a circadian variation in STEMI incidence but there was no evidence of a circadian variation in MI size based on the time of symptom onset measured by CK but half of the patients in this study received thrombolysis or were not revascularized.

All the above previous clinical studies have the limitations of using cardiac enzymes to measure peak MI size, with 6 out of 9 studies using creatine kinase (CK). CK has a high sensitivity to detect myocardial necrosis but has a low specificity [32]. Furthermore, cardiac enzyme levels in general are dependent on the timing of performing the test and some of these studies were retrospective studies [2], [6], [7] and therefore it was highly unlikely that the cardiac enzymes were performed at pre-specified times. Furthermore the choice (thrombolysis versus PPCI) [33] and the success of reperfusion strategy can influence the release of cardiac enzymes into the blood stream. CMR is considered the gold standard for the accurate quantification of acute MI size [21], [22], [23]. CMR provides additional information on the AAR, and it was difficult to account for that in the previous studies using cardiac enzymes alone. Reiter et al. [3] only included a subgroup of patients with CMR (n = 45) and CK was used to assess for a circadian variation in MI size for their whole cohort. Our study is the first to use CMR to measure acute MI size as a percentage of the AAR and explore the circadian rhythm according to the time of onset of symptoms and therefore we believe that our findings are more robust that previous studies. We have found that there was a 24-hour circadian variation in acute MI size depending on time of symptom onset in a subset of patients entering RCTs in the current era in a three-centre European (England, Italy and Spain) collaborative study. We did not find any significant circadian variation in a 12-hour cycle as previously shown [1] after adjusting for confounders. Our findings are consistent with the current belief that the circadian clocks are composed of proteins that generate self-sustaining, transcriptionally-based mechanisms of positive and negative feedback loops with a free-running period of approximately 24 h [34] rather than 12 h. There are several other factors that can affect MI size in the clinical setting (pre-infarct angina, onset to balloon time, co-morbidities, TIMI flow pre- and post-PCI and collateral flow) and failure to carefully adjust for these confounders may explain the conflicting findings between those from our study, Reiter et al. [3], Seneviratna et al. [6] and Mahmoud et al. [7] and those from Suarez-Barrientos et al. [1], Fournier et al. [4]and Ari et al. [5]. Furthermore, as a result of these multiple confounders of MI size, the circadian variation in MI size may not be clinically relevant for most patients. The recent large study by Mahmoud et al. [6] showed no impact on 1-year mortality. Our study showed that the extent of variation in MI size by CMR did not significantly influence acute LVEF and the likely explanation is that the difference between the peak and nadir MI size of 10.4% of the AAR was too small to affect the LV volumes significantly for the cohort as a whole but would likely be important for those with a large AAR. Our sample size was too small to only look at the circadian variation of LVEF in those patients with a large AAR and warrants further investigation in future studies AAR.

### 4.1 Limitations

This was a retrospective study and patient level data was obtained from 4 RCTs and therefore may not be a true representation of the STEMI cohort presenting to hospital. However, this is representative of patients entering RCTs in the current era. The sample size in the primary analysis group was < 50% of the original cohort but this was similar to the sample reported by Reiter et al. (n = 165) [3]. Patients were randomized to an intervention arm or placebo but we have adjusted for these factors in our analysis. We only included patients from one continent unlike the study by Ammirati et al. [19] but despite this, the time zone geographically may have some subtle differences among these 3 countries and we did not account for that. The modality used to quantify the AAR by CMR was different in the 4 studies and the contrast agents used for late gadolinium enhancement were varied as shown in Table 1. We did not have clinical outcomes on these patients. Our sample size was small and the cardioprotective therapies in the 4 RCTs were different. Therefore we were not able to assess whether the effectiveness of cardioprotective therapies also had a circadian dependence in this subset of patients depending on the time of day and this needs to be assessed in future larger studies.

## 5 Conclusions

We have shown that a circadian variation in acute MI size assessed by CMR according to the time of symptom onset exists in a specific group of patients, after adjusting for confounders of MI size. The largest and smallest MI size occurred in those patients with symptom onset between 00:00 to 01:00 and 12:00 to 13:00, respectively. This was associated with a non-significant circadian variation in acute LVEF. The circadian dependence in acute MI size was only observed in a subset of patients entering RCTs. This needs to be taken into account to make sure that the patients are adequately balanced in the 4 quartiles of time of onset of symptoms when designing future RCTs aiming to reduce MI size. Whether this would impact on the effectiveness of the cardioprotective therapies needs to be assessed in future larger studies.

## Conflicts of interest

None.

## Figures and Tables

**Table 1:** Details of the 4 RCTs with patient level data included in this study.

Studies	Country/years	Intervention	Patients in study	Timing of CMR	Area-at-risk	Acute MI size	Outcome
Ludman [27]	UK/2007–2009	Erythropoietin	51	1–6 days	Endocardial surface area	10 min after 0.2 mmol/kg Dotarem,	Erythropoietin failed to reduce MI size
Crimi [28]	Italy/2009–2011	Remote ischemic conditioning of the lower limbs	100	3–5 days	T2-weighted imaging	15 min after 0.2 mmol/kg Magnevist	Reduction in enzymatic MI size in the intervention arm. Under-powered to see a difference in MI size by CMR
Garcia-Dorado [30]	Spain/2008–2011	Adenosine	201	2–7 days	T2-weighted imaging	10 min after 0.2 mmol/kg of Magnevist	No difference in acute MI size in the whole cohort
White [29]	UK/2011–2012	Remote ischemic conditioning of the upper limb	197	3–6 days	T2 mapping	10 min after 0.1 mmol/kg of Dotarem	27% reduction in MI size by CMR in the intervention arm

MI: myocardial infarct; CMR: cardiovascular magnetic resonance.

**Table 2:** Patients' characteristics for the whole cohort.

	Total number of patients (n = 376)
Trials	
Ludman [27]	40 (11%)
Crimi [28]	76 (20%)
Garcia-Dorado [30]	177 (47%)
White [29]	83 (22%)
Randomization	
Intervention	191 (51%)
Placebo	185 (49%)
Age	59 ± 12
Sex — male	193 (51%)
Risk factors	
Smoking	221 (59%)
Hypertension	166 (44%)
Dyslipidemia	117 (31%)
Diabetes Mellitus	55 (15%)
Family history of CAD	72 (19%)
Pre-infarct angina	108 (29%)
Onset to balloon time/min	215 ± 97
Artery involved	
LAD	211 (56%)
RCA	71 (19%)
Cx	94 (25%)
TIMI flow pre-PPCI	
0	365 (97%)
1	11 (3%)
Rentrop collateral flow	
0	287 (76%)
1	61 (16%)
2	28 (7%)
TIMI flow post-PPCI	
1	1 (1%)
2	43 (11%)
3	330 (88%)
CMR details	
LVEDV	154 ± 36
LVESV	78 ± 31
LVEF	50 ± 11
MI size/%LV	22 ± 11
AAR/%LV	33 ± 12
MI size/%AAR	64 ± 21
MVO/%	111 (53%)

CAD: coronary artery disease; LAD: left anterior descending artery; RCA: right coronary artery; Cx: circumflex artery; TIMI: Thrombolysis In Myocardial Infarction; PPCI: primary percutaneous coronary intervention; LVEDV: left ventricular end diastolic volume; LVESV: left ventricular end systolic volume; LVEF: left ventricular ejection fraction; AAR: area at risk; LV: left ventricle; MVO: microvascular obstruction.

**Table 3:** Patients' characteristics for those in the primary analysis compared to those not in the primary analysis.

	Patients not in primary analysis n = 207	Patients in primary analysis n = 169	*p* value
Trials			
Ludman [27]	12 (30%)	28 (70%)	< 0.001
Crimi [28]	39 (51%)	37 (49%)	
Garcia-Dorado [30]	131 (74%)	46 (26%)	
White [29]	25 (30%)	58 (70%)	
Randomization			
Intervention	107 (56%)	84 (44%)	0.39
Placebo	100 (54%)	85 (46%)	
Age	58 ± 12	59 ± 11	0.16
Sex — male	80 (42%)	113 (58%)	< 0.001
Risk factors			
Smoking	134 (61%)	87 (39%)	0.006
Hypertension	91 (55%)	75 (45%)	0.51
Dyslipidemia	62 (53%)	55 (47%)	0.33
Diabetes mellitus	30 (55%)	25 (45%)	0.52
Family history of CAD	41 (57%)	31 (43%)	0.41
Onset to balloon time/min	235 ± 118	192 ± 64	< 0.001
Artery involved			
LAD	114 (54%)	97 (46%)	0.36
RCA	31 (44%)	40 (56%)	0.02
Cx	62 (66%)	32 (34%)	0.009
CMR details			
LVEDV	155 ± 39	153 ± 33	0.51
LVESV	81 ± 34	75 ± 25	0.56
LVEF	49 ± 11	52 ± 11	0.054
MI size/%LV	22 ± 12	22 ± 10	0.95
AAR/%LV	34 ± 13	32 ± 11	0.25
MI size/%AAR	63 ± 20	66 ± 21	0.08
MVO/%	111 (53%)	98 (47%)	0.23

CAD: coronary artery disease; LAD: left anterior descending artery; RCA: right coronary artery; Cx: circumflex artery; TIMI: Thrombolysis In Myocardial Infarction; PPCI: primary percutaneous coronary intervention; LVEDV: left ventricular end diastolic volume; LVESV: left ventricular end systolic volume; LVEF: left ventricular ejection fraction; AAR: area at risk; LV: left ventricle; MVO: microvascular obstruction.

**Table 4:** Summary of clinical studies investigating onset of symptoms and circadian variation of MI size in STEMI.

Studies	Country/years	Patients in study	Surrogate for MI size	Outcome
Suarez-Barrientos [1]	Single centre — Spain	811	CK TnI	Peak MI size between 06:00 and noon
Reiter [3]	Single centre — United States	165	CK	Peak MI size 01:00 onset of ischemia and 05:00 onset of reperfusion
Arroyo-Ucar [20]	Single centre — Spain	108	TnI	Peak MI size between 00:00 and 12:00
Fournier [4]	Single centre — Switzerland	353	CK	Peak MI size between 00:00 and 05:59.
Ammirati [19]	Multicentre — Italy, Scotland, and China	1099	CK	Peak MI incidence from 06:00 to noon. No clear-cut circadian dependence of MI size
Fournier [2]	Multicentre — Switzerland	6223	CK	Peak MI size at 23:00, whereas the nadir MI size was at 11:00. Risk of death from STEMI was the highest at 00:00 and lowest at 12:00
Seneviratna [6]	Multicentre — Singapore	6710	CK	Peak MI size and incidence of acute heart failure from midnight to 06:00 and nadir from 06:00 to noon
Mahmoud [7]	Multicentre — Netherlands	6799	CK	Peak MI size around 03:00 and nadir around 11:00
Ari [5]	Single centre — Turkey	252	CK-MB	Peak MI size and poor LV function by echocardiography occurred in the 06:00–noon period

CK: creatine kinase; TnI: troponin I; CK-MB: creatine kinase-myocardial band; MI: myocardial infarction; LV: left ventricle.
